# Socio-behavioral determinants of undiagnosed type 2 diabetes in middle-aged adults: a cross-sectional analysis

**DOI:** 10.3389/fpubh.2025.1735170

**Published:** 2026-01-06

**Authors:** Amani S. Alrossies, Nawal Alsubaie, Zafar Ali Shah, Muhammad Ilyas, Ijaz Habib, Gauhar Saddique, Syed Muzammil Shah

**Affiliations:** 1 Department of Pharmacy Practice, College of Pharmacy, Princess Nourah Bint Abdulrahman University, Riyadh, Saudi Arabia; 2 Department of Agricultural Chemistry & Biochemistry, The University of Agriculture, Peshawar, Peshawar, Khyber Pakhtunkhwa, Pakistan; 3 Department of Medicinal Chemistry, College of Pharmacy, University of Minnesota, Minneapolis, MN, United States; 4 Institute of Public Health and Social Sciences, Khyber Medical University, Peshawar, Khyber Pakhtunkhwa, Pakistan; 5 United Nations World Food Programme, Peshawar, Khyber Pakhtunkhwa, Pakistan; 6 Food and Agriculture Organization of the United Nations, Peshawar, Pakistan; 7 Department of Cardiology, Lady Reading Hospital Peshawar, Peshawar, Khyber Pakhtunkhwa, Pakistan

**Keywords:** body mass index, middle-aged adults, physical activity, risk screening, smoking, undiagnosed diabetes

## Abstract

**Background:**

Late diagnosis of type 2 diabetes mellitus causes preventable complications and higher morbidity. Obesity, inactivity, poor diet, and smoking increase the risk of diabetes; however, their relationship with undiagnosed diseases in middle-aged adults requires further clarification.

**Methods:**

Between January and June 2024, we enrolled 200 adults aged 35–45 years from outpatient clinics. The staff measured the height, weight, and body mass index of each participant. Participants completed the International Physical Activity Questionnaire and Mediterranean Diet Adherence Screener and reported their smoking histories. After overnight fasting, we measured the fasting blood glucose and HbA1c levels. Undiagnosed diabetes was defined according to the 2024 American Diabetes Association criteria (FBG ≥ 126 mg/dL or HbA1c ≥ 6.5%) in patients without a prior diagnosis. Statistical analyses included independent *t*-tests, chi-square tests, and multivariable logistic regression.

**Results:**

Undiagnosed diabetes was found in 29 participants (14.5, 95% CI 9.8–19.2). Those with undiagnosed diabetes had a higher body mass index (30.6 ± 2.6 vs. 27.6 ± 3.4 kg/m^2^, *p* < 0.001), lower physical activity prevalence (10.3% vs. 21.6%, *p* < 0.018), and higher smoking rates (48.3% vs. 26.3%, *p* < 0.024). After adjusting for physical activity, smoking, and diet quality, body mass index remained independently associated with undiagnosed diabetes (OR 1.36 per kg/m^2^, 95% CI 1.16–1.70, *p* < 0.001). Physical activity level (OR 1.54 per category decrease, 95% CI 1.30–1.92, *p* < 0.024) and smoking (OR 1.33, 95% CI 1.13–1.66, *p* < 0.005) were also independently associated with undiagnosed status. Model discrimination was good (area under the curve 0.74, 95% CI 0.67–0.81), with a 93.3% negative predictive value.

**Conclusion:**

Approximately one in seven middle-aged adults in outpatient settings has undiagnosed type 2 diabetes. A higher body mass index, lower physical activity, and smoking were independently associated with an undiagnosed status. These readily assessable socio-behavioral factors should inform screening strategies and risk assessments in primary care.

## Introduction

Between 30 and 50% of diabetes cases in some populations remain undiagnosed (1, 2). Individuals with undiagnosed diabetes are at an increased risk of cardiovascular disease, microvascular complications, and early death; however, early diagnosis can prevent many adverse outcomes (3, 4).

Adults aged 35–45 years needs particular attention for diabetes screening. Metabolic dysfunction begins during this period, but symptoms are often absent or subtle, creating an opportunity for early intervention before complications develop. Most screening guidelines rely on age and body mass index cutoffs; however, the addition of socio-behavioral markers could enhance detection rates and clinical utility (5, 6).

Obesity, sedentary behavior, poor diet quality, and tobacco use increase the risk of diabetes (7, 8). Prior research has primarily examined these factors in individuals with diagnosed diabetes or prediabetes, leaving gaps in understanding what distinguishes those with undiagnosed disease from healthy individuals (9, 10). Identifying these distinctions could sharpen screening tools and improve targeted interventions.

Sedentary behavior causes insulin resistance and metabolic dysfunction; however, physicians rarely assess physical activity during routine clinical examinations (11, 12). Smoking also increases the risk of diabetes, although its association with a delayed diagnosis requires further investigation. The burden of undiagnosed diabetes is particularly pronounced in the low- and middle-income countries of South Asia, where healthcare infrastructure remains limited and awareness of metabolic disorders is inadequate (13, 14).

In Khyber Pakhtunkhwa province, Pakistan, a region with a population exceeding 30 million, epidemiological surveillance data suggest that lifestyle changes accompanying rapid urbanization have contributed to rising obesity and sedentary behavior among middle-aged adults. However, systematic screening for diabetes in outpatient settings remains inconsistent, and many individuals with asymptomatic hyperglycemia are never identified during routine clinical encounters. This gap between disease prevalence and detection rates underscores the need for locally relevant, evidence-based screening strategies that leverage readily available clinical and behavioral data. Our study, conducted in Peshawar-based tertiary care facilities, addresses this regional need by examining the socio-behavioral determinants of undiagnosed diabetes in a population experiencing rapid health system transition (1, 15).

This study aimed to (1) estimate the prevalence of undiagnosed type 2 diabetes among middle-aged outpatients, (2) identify which socio-behavioral factors (body mass index, physical activity, diet quality, and smoking) were independently associated with undiagnosed diabetes status, and (3) evaluate whether these factors could support clinical risk stratification. We hypothesized that modifiable socio-behavioral factors would show independent associations with undiagnosed diabetes, suggesting practical targets for screening and prevention.

## Methods

### Study design and setting

From January to June 2024, we conducted a cross-sectional study at outpatient clinics of tertiary care hospitals in Peshawar, Khyber Pakhtunkhwa, Pakistan. The Institutional Ethics Committee approved the study protocol (Protocol #2024-DM-001). All procedures adhered to the principles of the Declaration of Helsinki, and the participants provided written informed consent before their enrolment.

### Participants

We recruited adults aged 35–45 years attending routine health checkups or preventive care visits through consecutive sampling.

#### Inclusion criteria

- Age 35–45 years
- No prior diabetes diagnosis
- Able to provide written informed consent
- Willing to complete study assessments

#### Exclusion criteria

- Diagnosed diabetes (any type)
- Pregnant or delivery within 6 months
- Hospitalization for acute illness within 4 weeks
- Conditions affecting glucose metabolism (Cushing’s syndrome, acromegaly)
- Taking medications that alter glucose (corticosteroids, antipsychotics, protease inhibitors)
- Physical or cognitive impairments preventing participation

### Sample size

To estimate 15% diabetes prevalence with ±5% precision at 95% confidence, allowing for 10% dropout, we required 196 participants. We enrolled 200 participants to ensure an adequate statistical power.

### Data collection

Trained staff collected data using standardized protocols during a single fasting visit. The staff recorded the age, sex, education level, occupation, and marital status of all participants.

#### Anthropometric measurements included

*Height*: measured to 0.1 cm precision using wall-mounted stadiometer without shoes.

*Weight*: measured to 0.1 kg precision on calibrated digital scale in light clothing.

*Body mass index*: calculated as weight (kg) ÷ height (m^2^).

#### Socio-behavioral measures

##### Physical activity

The International Physical Activity Questionnaire Short Form (IPAQ-SF) was used to measure the weekly metabolic equivalent (MET) minutes (10). The activities were classified as follows:

*Low*: <600 MET-min/week.

*Moderate*: 600–3,000 MET-min/week.

*High*: >3,000 MET-min/week.

##### Diet quality

A 5-point Mediterranean Diet Adherence Screener scored dietary patterns from 1 (poorest) to 5 (excellent), emphasizing fruits, vegetables, whole grains, and healthy fats (16).

##### Smoking

Participants reported their current smoking status (yes/no) based on cigarette use within the last 30 days. Pack-years were calculated for current and former smokers.

##### BMI classification

Body mass index was classified according to the World Health Organization criteria: underweight (<18.5 kg/m^2^), normal weight (18.5–24.9 kg/m^2^), overweight (25.0–29.9 kg/m^2^), and obese (≥30.0 kg/m^2^). For Asian populations, screening was recommended at BMI ≥ 23 kg/m^2^ per WHO guidelines (15).

### Laboratory testing

After an 8-h overnight fast (water permitted). The hospital’s certified laboratory performed the following tests.

#### Fasting blood glucose

Glucose oxidase method (coefficient of variation <2.5%).

#### HbA1c

High-performance liquid chromatography with NGSP certification (coefficient of variation <2.0%).

### Outcome definition

Undiagnosed type 2 diabetes was defined using the 2024 American Diabetes Association criteria as follows:

Fasting blood glucose ≥126 mg/dL (7.0 mmol/L), OR.

HbA1c ≥ 6.5% (48 mmol/mol) in participants without a prior diagnosis of diabetes.

We informed the participants who met the criteria and referred them for confirmatory testing and clinical management of the disease.

### Statistical analysis

Data were analyzed using Python 3.9 (Python Software Foundation) and SPSS version 28.0 (IBM Corp., Armonk, NY, USA). Statistical significance was set at *p* < 0.05.

#### Descriptive statistics

Continuous variables are presented as mean ± standard deviation (SD) or median with interquartile range based on distribution normality. Categorical variables are presented as frequencies and percentages.

#### Bivariate analyses

Groups were compared using independent *t*-tests for normally distributed continuous variables, Mann–Whitney U tests for non-normal distributions, and chi-square or Fisher’s exact tests for categorical variables.

#### Multivariable logistic regression

Binary logistic regression was used to model the outcomes of undiagnosed diabetes. Predictor variables were selected based on (1) clinical significance, (2) univariate association (*p* < 0.20), and (3) absence of multicollinearity (variance inflation factor <3.0). The final multivariable model included the following variables: body mass index (continuous, kg/m^2^), physical activity (ordinal: low, moderate, and high), smoking status (binary: yes/no), and diet quality score (continuous, 1–5 scale). Age and sex were assessed but not retained owing to a lack of statistical significance. The model fit was evaluated using the Hosmer-Lemeshow test (*p* > 0.05, indicating an adequate fit). Discrimination was quantified using the area under the receiver operating characteristic curve (AUC). The results are presented as odds ratios (OR) with 95% confidence intervals (CI).

#### Model performance

Sensitivity, specificity, positive predictive value (PPV), negative predictive value (NPV), and overall accuracy were calculated at the optimal cutoff determined by the Youden index.

#### Data quality

Data were independently entered by two staff members, and discrepancies were resolved by consensus. Range and logical consistency checks were performed to verify data quality. Missing data were <2% for all variables and were analyzed under a missing-at-random assumption without imputation. All statistical codes are available in the Supplementary materials for reproducibility purposes.

## Results

### Participant characteristics

All 200 participants completed the study. The mean age was 40.0 ± 2.7 years, and 102 (51.0%) patients were male. The descriptive statistics for the continuous variables are presented in Table 1.

**Table 1:** Baseline characteristics of study participants (*N* = 200).

Variable	Mean ± SD	Range
Age (years)	40.0 ± 2.7	35–45
Body mass index (kg/m^2^)	28.1 ± 3.4	19.5–35.5
Diet quality score (1–5)	3.1 ± 1.1	1–5
Fasting blood glucose (mg/dL)	110.4 ± 14.9	80–144.9
HbA1c (%)	5.90 ± 0.59	4.5–7.18

### Prevalence of undiagnosed diabetes

Undiagnosed type 2 diabetes was identified in 29 of the 200 participants (14.5, 95% CI 9.8–19.2%). Among these cases, 17 (58.6%) met the diagnostic criteria based on both elevated fasting blood glucose and HbA1c levels, eight (27.6%) based on HbA1c levels alone, and four (13.8%) based on fasting blood glucose levels alone. Using only one test would have missed 27.6% or 13.8% of cases, respectively, demonstrating the complementary value of both markers in PTC diagnosis.

The prevalence was higher in women (18.4%) than in men (10.8%); however, this difference was not statistically significant (*p* = 0.12), suggesting that screening should target both sexes equally in this age group.

### Comparison by diabetes status

Table 2 presents the baseline characteristics stratified by diabetes status. Participants with undiagnosed diabetes differed significantly from those without diabetes in several domains.

**Table 2:** Comparison of characteristics by diabetes status.

Characteristic	Total (*N* = 200)	No diabetes (*n* = 171)	Undiagnosed diabetes (*n* = 29)	*p*-value
Age (years), mean ± SD	40.0 ± 2.7	40.1 ± 2.7	39.5 ± 2.5	0.28
Male sex, *n* (%)	102 (51.0)	91 (53.2)	11 (37.9)	0.12
BMI (kg/m^2^), mean ± SD	28.1 ± 3.4	27.6 ± 3.4	30.6 ± 2.6	**<0.001**
Physical activity, *n* (%)				0.018
Low	60 (30.0)	52 (30.4)	8 (27.6)	
Moderate	100 (50.0)	82 (48.0)	18 (62.1)	
High	40 (20.0)	37 (21.6)	3 (10.3)	
Diet quality score, mean ± SD	3.1 ± 1.1	3.2 ± 1.0	3.0 ± 1.1	0.39
Current smoking, *n* (%)	50 (25.0)	41 (24.0)	9 (31.0)	0.42
Fasting blood glucose (mg/dL), mean ± SD	110.4 ± 14.9	106.3 ± 11.7	134.7 ± 5.3	**<0.001**
HbA1c (%), mean ± SD	5.90 ± 0.59	5.74 ± 0.46	6.86 ± 0.21	**<0.001**

*p*-values from independent *t*-tests (continuous) or chi-square tests (categorical). Bold indicates *p* < 0.05.

### Physical activity and diabetes prevalence

**Figure 1:**
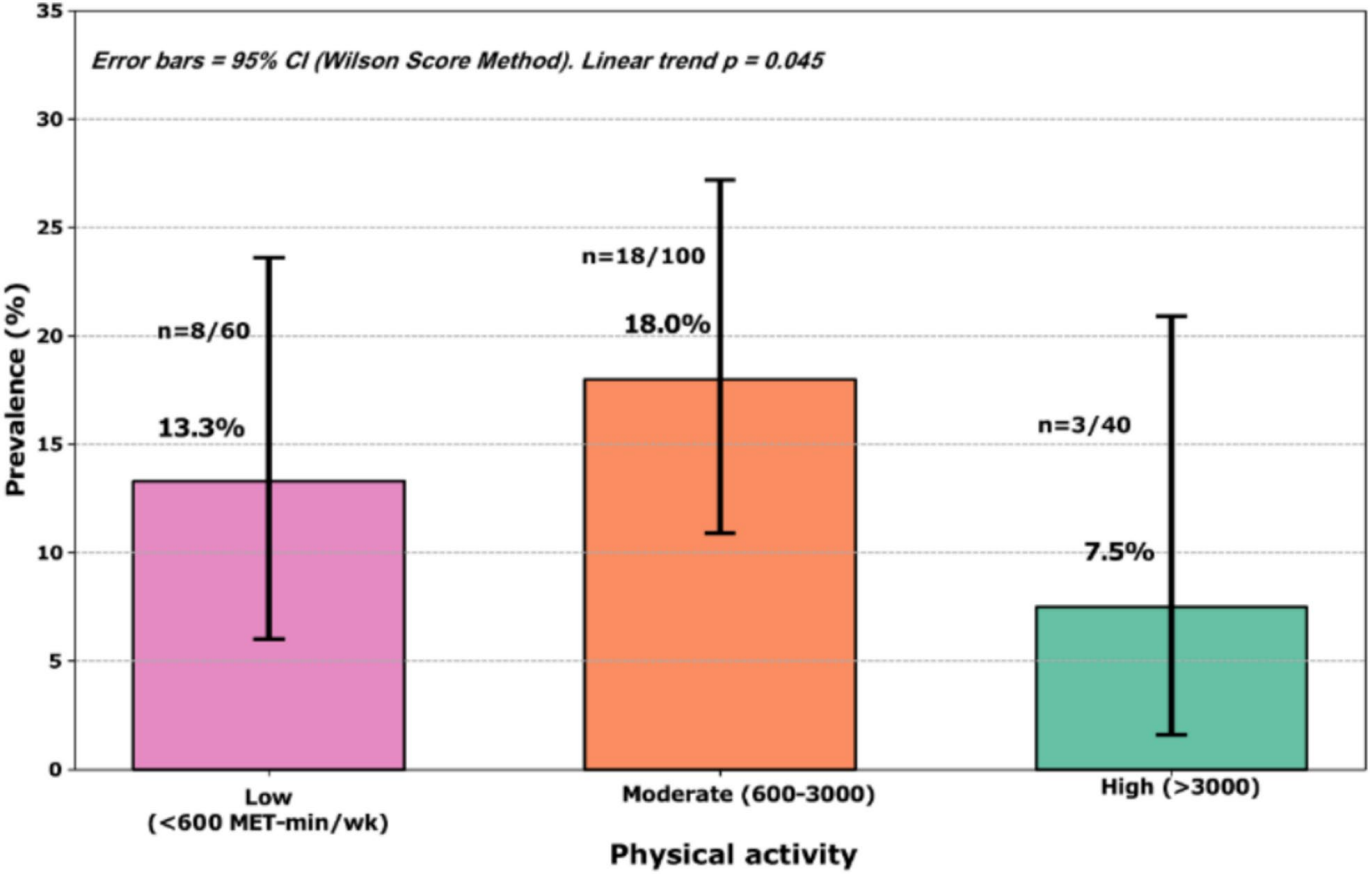
Prevalence of undiagnosed type 2 diabetes according to physical activity levels. Bar chart showing the prevalence of diabetes in the three activity categories. Error bars represent 95% confidence intervals. The numbers indicate the number of cases/total participants. Linear trend test *p* = 0.045.

Physical activity showed a dose–response relationship with undiagnosed diabetes (Figure 1). Among participants with low activity, the prevalence was 13.3% (8/60), among those with moderate activity, it was 18.0% (18/100), and among highly active participants, it was 7.5% (3/40).

Linear trend analysis confirmed a statistically significant dose–response relationship (*p* = 0.04), indicating that increased physical activity provides incremental protection against undiagnosed diabetes mellitus. Notably, highly active participants had less than half the prevalence of undiagnosed diabetes compared with those with low activity.

### Body mass index distribution

**Figure 2:**
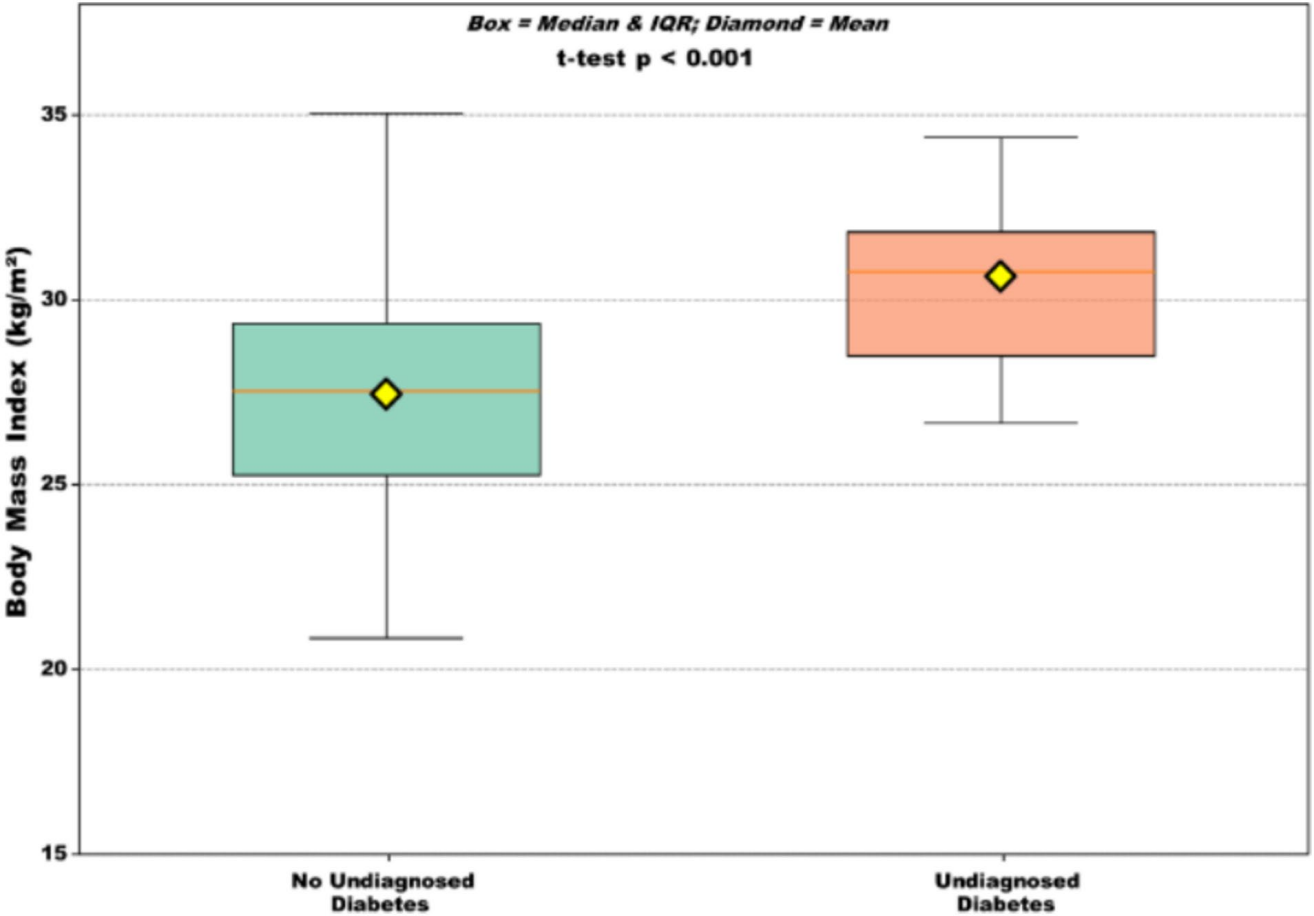
Body mass index distribution according to undiagnosed diabetes status. Box plots show median (horizontal line), interquartile range (box), and range (whiskers). Yellow diamonds represent the mean values. Participants with undiagnosed diabetes had a significantly higher BMI (30.6 ± 2.6 kg/m^2^) than those without (27.6 ± 3.4 kg/m^2^) (*p* < 0.001).

The body mass index was significantly higher in participants with undiagnosed diabetes than in those without (Figure 2).

Among those with undiagnosed diabetes, 58.6% (17/29) had a BMI ≥ 30 kg/m^2^, whereas 25.7% (44/171) of those without diabetes had this BMI level (*p* < 0.001).

### Multivariable analysis

**Figure 3:**
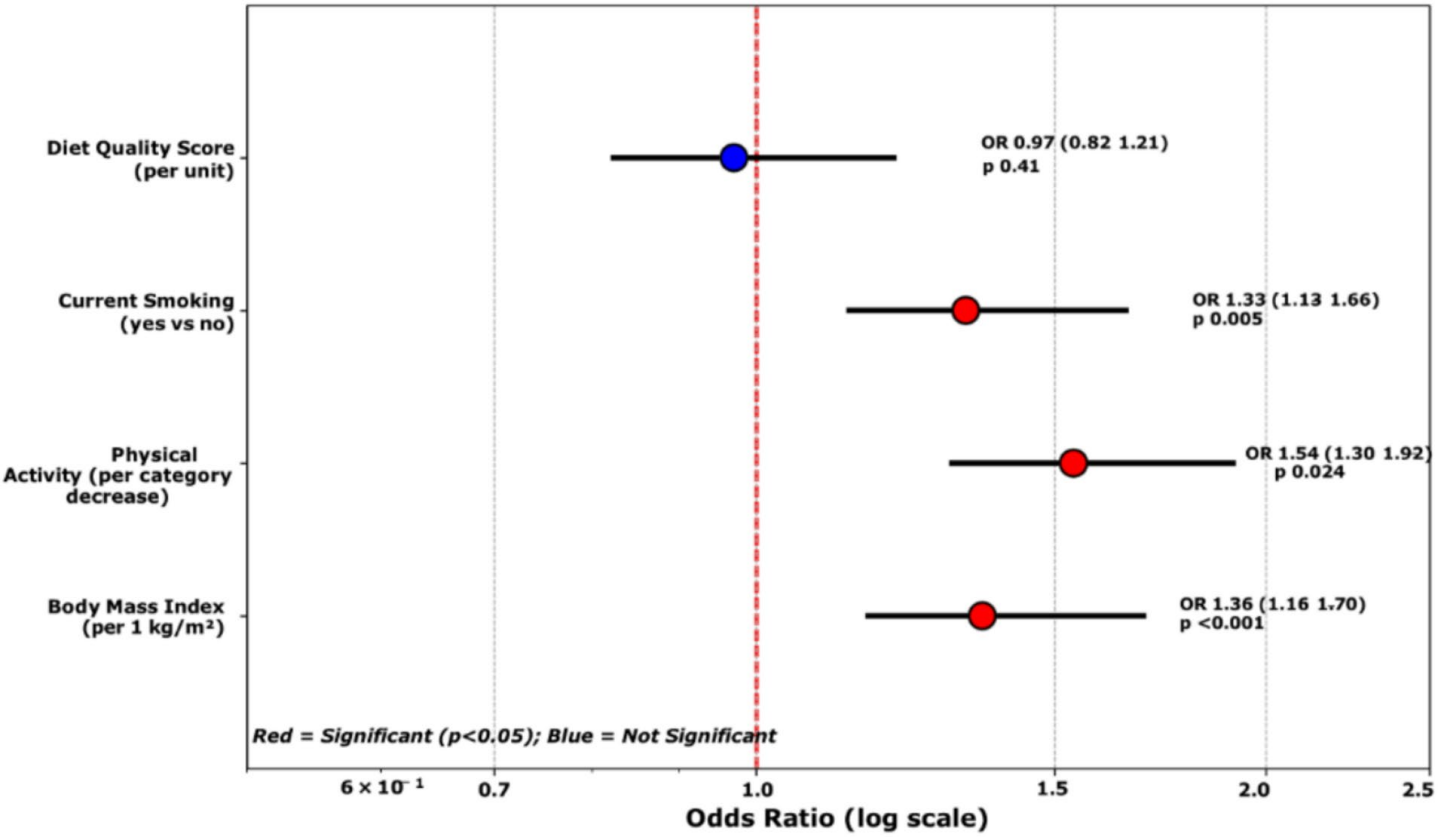
Forest plot of the multivariate logistic regression results. Odds ratios and 95% confidence intervals for the predictors of undiagnosed diabetes. BMI expressed per 1 kg/m^2^ increase; physical activity per category decrease (high → moderate → low); smoking (current vs. non-smoker); and diet score per 1-point increase. The vertical dashed line indicates OR = 1 (no association). Points to the right of the line indicate increased odds, and points to the left indicate protective effects. The only protective finding was the diet score (odds ratio <1.0), which was not statistically significant.

Table 3, Figure 3 present the results of the multivariable logistic regression analysis. After adjusting for physical activity, smoking, and diet quality score, body mass index remained the strongest predictor, with each 1 kg/m^2^ increase associated with 36% higher odds of undiagnosed diabetes. Physical activity showed a dose–response relationship, with each category decrease increasing the odds by 54%. Current smoking was independently associated with a 33% higher risk. Diet quality score was not statistically significantly associated (*p* = 0.41) but was retained in the final model for a comprehensive evaluation of socio-behavioral factors.

**Table 3:** Multivariable logistic regression for undiagnosed type 2 diabetes.

Predictor	Odds ratio	95% CI	*p*-value
Body mass index (per 1 kg/m^2^)	1.36	1.16–1.70	<0.001
Physical activity (per category decrease)	1.54	1.30–1.92	0.024
Smoking (yes vs. no)	1.33	1.13–1.66	0.005
Diet quality score (per unit)	0.97	0.82–1.21	0.41

All four predictor variables were retained in the final model despite non-significant diet score (*p* = 0.41) for comprehensive evaluation of socio-behavioral factors.

### Model performance

All four predictor variables were retained in the final model for a comprehensive evaluation of socio-behavioral factors despite the non-significant diet score association.

Model discrimination was good, with an area under the curve (AUC) of 0.74 (95% CI 0.67–0.81), indicating an acceptable ability to distinguish between participants with and without undiagnosed diabetes.

At the optimal cutoff (predicted probability 0.15), the model achieved the following results, sensitivity, 65.5% (95% CI 45.7–82.1%), specificity, 73.7% (95% CI 66.4–80.2%), positive predictive value, 28.4% (95% CI 18.9–39.5%), negative predictive value, 93.3% (95% CI 88.1–96.8%) and overall accuracy, 72.5% (95% CI 65.7–78.7%).

The low positive predictive value reflects the relatively low prevalence of undiagnosed diabetes in the study population; however, the high negative predictive value is clinically useful, correctly identifying 93% of truly negative cases. Individuals with low predicted risk profiles may safely defer glucose testing.

## Discussion

We identified undiagnosed type 2 diabetes in 14.5% of middle-aged adults who attended outpatient clinics. Body mass index, physical inactivity, and smoking were independently associated with undiagnosed status. These findings have direct implications for diabetes screening and risk assessment in primary healthcare.

### Prevalence findings

The prevalence of undiagnosed diabetes in our study (14.5%) aligns with global estimates of 30–50% (2) and is consistent with recent US data showing approximately 20% of adults with diabetes remain undiagnosed (17). More than one-quarter of undiagnosed cases (23.6%) had elevated HbA1c levels with normal fasting glucose levels, confirming the complementary value of both markers, consistent with the American Diabetes Association’s guidelines (18).

Unlike some studies that have shown a higher prevalence in men, we found no sex difference in the prevalence. This may reflect local healthcare patterns or population characteristics and suggests that screening should target both sexes equally in this age group.

### Body mass index as the strongest association

Our finding that each 1 kg/m^2^ increase in BMI was associated with 36% higher odds of undiagnosed diabetes (OR 1.36, 95% CI 1.16–1.70) is consistent with meta-analytic evidence (7, 19). The strong association between obesity and insulin resistance is well-established (20), with adipose tissue dysfunction playing a central mechanistic role. No participants with a body mass index (BMI) below 25 kg/m^2^ had undiagnosed diabetes, whereas 27.9% of those with a body mass index ≥30 kg/m^2^ did. These findings support the current screening guidelines recommending testing at a body mass index of ≥25 kg/m^2^ (≥23 kg/m^2^ for Asian populations).

The risk increased continuously across the entire body mass index spectrum, rather than only above specific thresholds. Healthcare systems should routinely measure body mass index and initiate glucose testing at the recommended levels.

The association between obesity and diabetes likely stems from both biological mechanisms (insulin resistance and beta-cell dysfunction) and healthcare system gaps (insufficient screening of overweight patients). Interventions must address both pathways.

### Physical activity’s protective relationship

Physical activity demonstrated a dose–response relationship with the prevalence of undiagnosed diabetes. The prevalence reached 18.0% among those with moderate activity and 7.5% among those with high activity levels. After adjusting for body mass index and other variables, each decline in activity level increased the odds by 54%, indicating substantial protection against regular activity (15).

Multiple mechanisms likely explain this relationship, including improved insulin sensitivity, enhanced glucose uptake by muscles, reduced visceral adiposity, and decreased systemic inflammation (11, 20). Moderate activity (compared to low activity) also showed protective effects, supporting the practical message for clinical counselling that some activity is better than none.

Current guidelines recommend 150 min of moderate-intensity activity weekly; however, physicians infrequently assess or counsel physical activity (15). A simple screening question “On how many days per week do you engage in 30 or more minutes of moderate physical activity?” could identify high-risk patients who require testing and lifestyle interventions (21).

### Smoking’s independent role

Smoking was independently associated with undiagnosed diabetes (OR 1.33), although with less strength than the body mass index (13). This modifiable risk factor has dual health implications: smokers face elevated cardiovascular and cancer risks, in addition to diabetes, amplifying the benefits of smoking cessation.

The mechanisms linking smoking and diabetes include insulin resistance, central fat accumulation, chronic inflammation, and beta-cell dysfunction (13). Smokers may also use preventive healthcare services less frequently, contributing to delayed diagnosis.

Incorporating diabetes screening into smoking cessation programs could enhance case detection during this “teachable moment” for health behavior changes. Similarly, diabetes prevention programs should provide smoking cessation support as a core component of their intervention (14, 22).

### Diet quality findings

The diet quality score was not significantly associated with undiagnosed diabetes (OR 0.97, *p* = 0.41). Several explanations merit consideration: (1) our brief dietary screening tool may miss important dietary patterns; (2) dietary effects may manifest over longer timeframes than those captured in this cross-sectional study; (3) body mass index may mediate dietary effects, masking direct associations; or (4) diet quality may genuinely have no independent effect on this population (23).

Detailed food frequency questionnaires may reveal stronger associations. Specific dietary components, such as sugar-sweetened beverages or ultra-processed foods, may demonstrate clearer relationships than overall dietary quality (24).

### Practical applications

Our findings suggest several actionable strategies.

#### Risk-based screening

Develop simple screening algorithms incorporating the body mass index, physical activity level, and smoking status. For example, automatically order glucose testing for adults with a body mass index ≥28 kg/m^2^ who also report low physical activity or current smoking in electronic health records.

#### Link screening to lifestyle programs

Connect positive screening results to evidence-based programs such as the Diabetes Prevention Program. Addressing obesity, inactivity, and smoking simultaneously may produce synergistic benefits compared with addressing each factor individually (25).

#### Routine assessment

Physical activity and smoking status were measured as standard vital signs alongside body mass index. Medical assistants should be trained to collect this information before the provider’s encounter.

#### Community-based screening

Body mass index, physical activity, and smoking can be assessed in nonclinical settings. Pharmacies and workplaces can implement screening programs and refer high-risk individuals to healthcare providers for diagnostic testing.

#### Public health messaging

Public health campaigns emphasizing the connections between obesity, sedentary lifestyle, smoking, and diabetes could increase population awareness and encourage screening participation and preventive behaviors (26–29).

The findings from this Peshawar-based cohort have direct relevance to resource-constrained healthcare systems in South Asia, where clinician time and laboratory capacities are limited. Risk-based screening using body mass index, physical activity, and smoking status, all assessable through brief clinical questioning, offers a pragmatic alternative to universal screening approaches that place an excessive burden on laboratory services. Countries such as Pakistan, Bangladesh, and India have prioritized diabetes detection as part of their noncommunicable disease control programs; however, implementation remains fragmented across primary, secondary, and tertiary care settings. Our model, with its high negative predictive value (93.3%), can reliably identify low-risk individuals who can safely defer glucose testing, thereby optimizing resource allocation in settings where HbA1c and fasting glucose testing capacity is limited (1, 15). The clustering of obesity, physical inactivity, and smoking in this cohort reflects broader patterns of health behavior changes in urbanizing South Asian populations (13). Public health campaigns in Pakistan and neighboring countries should emphasize the interconnected nature of these risk factors and leverage existing infrastructure, such as hypertension or family planning clinics, to opportunistically screen middle-aged adults for undiagnosed diabetes.

### Strengths and limitations

#### Strengths

This study employed rigorous laboratory diagnosis using both fasting glucose and HbA1c, validated questionnaires for behavioral assessment, appropriate multivariable statistical modelling, an adequate sample size for prevalence estimation, and minimal missing data (<2%).

#### Limitations

The cross-sectional design precludes causal inference, and only longitudinal studies can establish temporal relationships. The single-center recruitment limits the generalizability of the findings to other healthcare settings or populations. Self-reported behavioral measures may suffer from social desirability bias, potentially attenuating true associations. We did not assess family history, socioeconomic status, psychosocial stress, or sleep duration, which are potential confounders. The sample size (*n* = 200, 29 cases) was insufficient to provide adequate statistical power for subgroup analysis or interaction testing.

We did not examine healthcare utilization patterns or screening barriers, both of which are likely to influence diagnosis timing. Cost-effectiveness analyses would clarify optimal screening strategies in resource-constrained settings. Our focus on middle-aged adults in outpatient clinics may not extend to younger or older populations or community-dwelling individuals who are not engaged in routine health care.

### Research priorities

#### Key next steps include

##### Prediction tool validation

Develop and validate a clinical prediction tool incorporating socio-behavioral factors, and then test it in prospective cohorts across diverse settings.

##### Implementation science

Conduct pragmatic trials to evaluate screening interventions embedded in routine care, such as automated glucose testing triggered by electronic health record risk scores.

##### Economic analysis

Compare the cost-effectiveness of risk-based and age-based screening strategies, considering detection yields and resource utilization.

##### Mechanism research

Clarify biological and healthcare system pathways linking socio-behavioral factors to delayed diagnosis using mediation analyses in large cohorts.

##### Intervention trials

Test whether lifestyle programs targeting high-risk individuals based on socio-behavioral profiles prevent progression to overt diabetes.

##### Health equity assessment

Examine whether socio-behavioral screening performs equitably across racial, ethnic, and socioeconomic groups to identify and address potential disparities.

## Conclusion

Approximately one in seven middle-aged adults in outpatient care settings has undiagnosed type 2 diabetes. A higher body mass index, lower physical activity, and smoking were independently associated with undiagnosed diabetes. These readily assessable socio-behavioral factors should be considered in screening decisions and risk stratification in primary care.

Clinicians should routinely assess body mass index, physical activity, and smoking status during preventive care visits and use these markers to guide glucose testing decisions. Public health initiatives that promote weight management, physical activity, and smoking cessation can prevent diabetes and facilitate its early detection.

Integrating simple socio-behavioral assessments into clinical workflows offers a practical approach to improve screening efficiency and effectiveness. Future studies should test and refine these risk-based strategies in real-world settings, considering cost-effectiveness and health equity.

## Data Availability

The original contributions presented in the study are included in the article/Supplementary material, further inquiries can be directed to the corresponding authors.
