# Supplementary material for: Socio-behavioral determinants of undiagnosed type 2 diabetes in middle-aged adults: a cross-sectional analysis
Source: Front Public Health. 2026 Jan 6;13:1735170. doi: 10.3389/fpubh.2025.1735170 (PMC12815826; doi:10.3389/fpubh.2025.1735170)
Supplement: Supplementary file 1 [file Table_1.docx]

**Supplementary Materials**

Additional file 1: Supplementary Table S1. Detailed demographic and clinical characteristics stratified by age groups and BMI categories.

Additional file 2: Supplementary Table S2. Univariate logistic regression analysis for all potential predictors of undiagnosed diabetes.

Additional file 3: Supplementary Table S3. Model performance metrics including sensitivity, specificity, and predictive values at various probability cut-points.

Additional file 4: Complete dataset (diabetes_study_dataset.csv) - De-identified individual participant data.
